# The cAMP/Protein Kinase A Pathway Regulates Virulence and Adaptation to Host Conditions in *Cryptococcus neoformans*

**DOI:** 10.3389/fcimb.2019.00212

**Published:** 2019-06-18

**Authors:** Mélissa Caza, James W. Kronstad

**Affiliations:** Michael Smith Laboratories, Department of Microbiology and Immunology, University of British Columbia, Vancouver, BC, Canada

**Keywords:** cAMP/PKA pathway, cryptococcus, nutrient sensing, RIM pathway, HOG pathway, titan cells, capsule, melanin

## Abstract

Nutrient sensing is critical for adaptation of fungi to environmental and host conditions. The conserved cAMP/PKA signaling pathway contributes to adaptation by sensing the availability of key nutrients such as glucose and directing changes in gene expression and metabolism. Interestingly, the cAMP/PKA pathway in fungal pathogens also influences the expression of virulence determinants in response to nutritional and host signals. For instance, protein kinase A (PKA) in the human pathogen *Cryptococcus neoformans* plays a central role in orchestrating phenotypic changes, such as capsule elaboration and melanin production, that directly impact disease development. In this review, we focus first on insights into the role of the cAMP/PKA pathway in nutrient sensing for the model yeast *Saccharomyces cerevisiae* to provide a foundation for understanding the pathway in *C. neoformans*. We then discuss key features of cAMP/PKA signaling in *C. neoformans* including new insights emerging from the analysis of transcriptional and proteomic changes in strains with altered PKA activity and expression. Finally, we highlight recent studies that connect the cAMP/PKA pathway to cell surface remodeling and the formation of titan cells.

## Introduction

**Figure 1:**
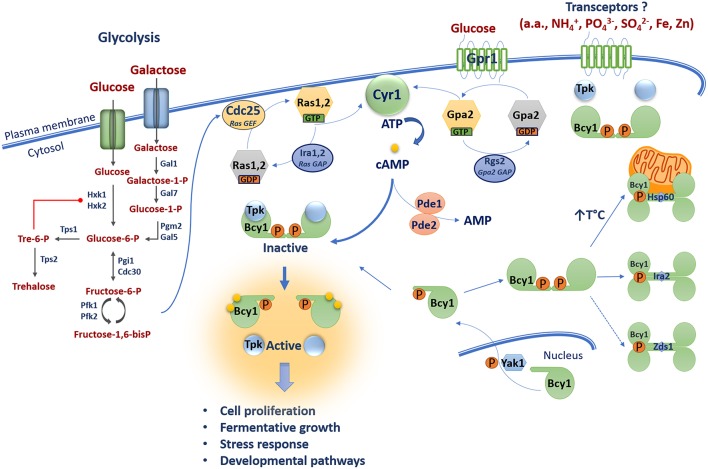
The cAMP/PKA pathway in *S. cerevisiae* is dually activated by glucose via one mechanism involving glycolysis and the Ras-cAMP pathway proteins, and a second pathway of glucose sensing via the G-protein coupled receptor Gpr1. Glycolysis generates fructose-1,6-bis-phosphate which activates the Ras nucleotide exchange factor Cdc25 which in turn activates the GTPases Ras1 and Ras2. These proteins are inactivated by the GAPs Ira1 and Ira2. GTP-bound Ras1 and Ras2 promote cAMP production via activation of the adenylyl cyclase Cyr1. Gpr1 senses external glucose and acts via the GTPase Gpa2 to stimulate adenylyl cyclase Cyr1 to produce cAMP. Elevated cAMP promotes the dissociation of the catalytic (Tpk1, 2, 3) and regulatory (Bcy1) subunits of PKA leading to activation of PKA and downstream signaling. The phosphodiesterases Pde1 and Pde2 control intracellular cAMP levels by degradation. The subcellular distribution of Bcy1 is regulated by Yak1-dependent phosphorylation and oligomerization of D/D domains of Bcy1 results in a tetrameric structure of allowing interactions with putative AKAPs (i.e., Hsp60, Ira2, and Zds1). Transceptor proteins mediate PKA activation independent of changes in cAMP levels in response to ammonium (NH_4_^+^), amino acids (a.a.), phosphate (PO_4_^3−^), sulfate (SO_4_^2−^), iron (Fe), and zinc (Zn).

The ability to rapidly adapt to changing external conditions is crucial for the survival and proliferation of microorganisms. This is particularly true for pathogenic microbes because they must make the transition from the environment to host conditions and mount an appropriate response to establish an infection. Challenging conditions in hosts include differences in nutrient availability, pH, oxygen levels, and temperature, as well as threats posed by the immune response. Signal transduction pathways play critical roles in mediating microbial adaptation. For example, in fungi, the availability of nutrients such as glucose is sensed through a number of mechanisms including the cyclic AMP/protein kinase A (cAMP/PKA) signal transduction pathway. The main components and functions of the cAMP/PKA pathway in nutrient sensing have been extensively characterized in the model yeast *Saccharomyces cerevisiae* (Figure 1). The framework of the pathway emerged from two lines of research in this yeast: (1) the identification of the signaling functions of the oncogenic mammalian Ras protein using yeast as a model system, and (2) the cAMP-induced phosphorylation of trehalase, an enzyme involved in trehalose mobilization in fungi (Powers et al., 1984; Thevelein, 1984). Integration of information from these lines of investigation led to the view that Ras proteins mediate intracellular glucose sensing in a glycolysis-dependent manner, whereas extracellular glucose is sensed through a G-protein coupled receptor (GPCR) system (Thevelein and de Winde, 1999). The glucose-sensing components in *S. cerevisiae* activate the adenylyl cyclase Cyr1 to produce cAMP that in turn activates PKA, a heterotetrameric protein comprised of two catalytic and two regulatory subunits. The catalytic subunits are encoded by the *TPK1-3* genes and the regulatory subunit is encoded by the *BCY1* gene (Toda et al., 1987a,b). To activate the enzyme, two molecules of cAMP bind to the regulatory subunits and trigger conformational changes that dissociate the complex, resulting in activation of the catalytic subunits of PKA for subsequent phosphorylation of substrates in various subcellular compartments. As discussed herein, a detailed view has emerged over the years of the complexities of cAMP/PKA signaling in yeast, and this information is useful for appreciating the contributions of the pathway to nutrient adaptation in other organisms, including fungal pathogens.

Interestingly, the mechanisms of adaptation to nutrient availability not only support proliferation but also can regulate the virulence of pathogens. The roles of the cAMP/PKA pathway in pathogenic fungus, *Cryptococcus neoformans*, provide a useful illustration of this phenomenon. In this fungus, the pathway influences key virulence traits associated with proliferation in vertebrate hosts including elaboration of a polysaccharide capsule, deposition of the pigment melanin in the cell wall, sensing of nutrients (glucose, amino acids, iron, phosphate), the response to stress (including pH), cell wall integrity, and the formation of an enlarged cell type called titan cells (Alspaugh, 2015; Esher et al., 2018; García-Rodas et al., 2018). The analysis of host adaptation by *C. neoformans* is important because of the global impact of this fungus (and the related *Cryptococcus gattii* species complex) on human health. Specifically, meningoencephalitis caused by these fungal species is one of the most prevalent invasive diseases in humans suffering from HIV/AIDS (Rajasingham et al., 2017). In fact, ~300,000 cases of cryptococcal meningoencephalitis occur per year with ~200,000 associated deaths. Remarkably, isolates of the *C. gattii* species complex also cause disease in immunocompetent people as demonstrated by an outbreak of cryptococcosis in the Pacific Northwest of North America (Bartlett et al., 2012). Cryptococcal infections are acquired by inhalation of fungal cells from environmental sources including trees, soil, and bird excreta. The fungal cells therefore face the challenge of shifting from conditions of lower temperature and limited nutrient availability in the environment to the warmer and distinct nutritional milieu in vertebrate hosts.

In this review, we first highlight aspects of cAMP/PKA signaling where specific findings in *S. cerevisiae* and mammalian cells inform and guide the analysis of pathway integration with virulence and host adaptation in *C. neoformans*. We then focus on aspects of cAMP/PKA signaling that specifically contribute to virulence mechanisms in *C. neoformans*. Several excellent reviews provide additional recent information on cAMP/PKA signaling and nutrient and host sensing by model fungi and fungal pathogens (Watkins et al., 2017; Sherrington et al., 2018; Steyfkens et al., 2018);(Rutherford et al., 2019; Van Ende et al., 2019).

## Mechanisms of Nutrient Sensing Mediated by the camp/pka Pathway in *S. cerevisiae*

### Pathway Activation and Connections With Glycolysis

Activation of the cAMP/PKA signaling pathway via glycolysis is a key aspect of the response of *S. cerevisiae* to external sugars (Figure 1). External fermentative sugars such as glucose and galactose enter the cell through hexose transporters (HXTs). Glucose is then phosphorylated by hexokinases (Hxk1 and Hxk2) to produce glucose-6-phosphate (Bisson and Fraenkel, 1983; Walsh et al., 1983). Similarly, galactose is phosphorylated by the galactokinase Gal1 to produce galactose-1-phosphate (Douglas and Condie, 1954; Schell and Wilson, 1977). This product is converted into glucose-1-phosphate by the galactose-1-phosphate uridyl transferase Gal7 and into glucose-6-phosphate by the phosphoglucomutase Pgm2/Gal5 (Segawa and Fukasawa, 1979; Boles et al., 1994). Glucose-6-phosphate can be converted into trehalose by the trehalose-6-phosphate synthases Tps1 and Tps2 (De Virgilio et al., 1993). Trehalose is a disaccharide of two glucose molecules and it serves as a carbohydrate storage molecule, as a stress protectant in yeast and fungi, and as a signaling molecule in plants (O'Hara et al., 2013). Trehalose-6-phosphate negatively regulates glycolysis by allosterically inhibiting Hxk1 and Hxk2 (Blázquez et al., 1993). Glucose-6-phosphate can also be further converted into fructose-6-phosphate by the phosphoglucose isomerases Pgi1 and Cdc30 (Dickinson and Williams, 1987; Dickinson, 1991). Fructose-6-phosphate is then converted into fructose-1,6-bisphosphate by phosphofructokinases Pfk1 and Pkf2 (Lobo and Maitra, 1983; Parmar et al., 1984). Importantly, recent work revealed that fructose-1,6-bisphosphate is the key product that connects glycolysis to the cAMP/PKA pathway. Specifically, fructose-1,6-bisphosphate is a potent activator of the RAS-cAMP pathway by acting on the highly conserved C-terminus of the guanine nucleotide exchange factor (GEF) Cdc25 (Peeters et al., 2017). The GTPases Ras1 and Ras2 are inactive when bound to guanosine diphosphate (GDP) and they became active when bound to guanosine triphosphate (GTP) through the action of the GEF Cdc25 (Broach, 1991; Jones et al., 1991). Conversely, these GTPases are negatively regulated by the GTPase activating proteins (GAPs) Ira1 and Ira2 that promote hydrolysis of GTP to GDP thus shifting Ras1 and Ras2 to the inactive GDP-loaded state (Tanaka et al., 1989, 1990). In their GTP-bound states, Ras1 and Ras2 promote cAMP production through activation of the adenylyl cyclase Cyr1 by interaction with its Ras associating (RA) domain (Broek et al., 1985; Kido et al., 2002).

Studies in *S. cerevisiae* also revealed that production of cAMP through the activity of Cyr1 is promoted by the glucose-sensing G-protein coupled receptor (GPCR) Gpr1 (Xue et al., 1998). GPCRs are highly conserved from yeast to humans and function via coupled heterotrimeric G proteins subunits (α, β, and γ). These receptors sense ligands such as sugars and pheromones, and undergo conformational changes that recruit the heterotrimeric G protein and promote GDP-GTP exchange on the Gα subunit and release the Gβγ complex (Gilman, 1987). Hence, upon glucose or sucrose sensing, Gpr1 interacts with the heterotrimeric Gα subunit Gpa2 and is believed to promote exchange of GDP-GTP because constitutive activation of Gpa2 stimulates the proliferation of a strain lacking both *RAS* genes in the presence of glucose (Xue et al., 1998; Lemaire et al., 2004). However, Gpa2 is unusual as it functions without typical Gβγ subunits (Harashima and Heitman, 2002). Instead, the Kelch proteins Gpb1 and Gbp2 are thought to associate with the GTPase Gpa2 to relieve inhibition of PKA, thereby bypassing adenylyl cyclase regulation (Peeters et al., 2006). Gpa2 was first discovered based on sequence similarity to mammalian Gα proteins (Nakafuku et al., 1988) and was shown to be involved in glucose-induced cAMP signaling (Colombo et al., 1998). Gpr1 was discovered in a two-hybrid interaction screen with Gpa2 (Yun et al., 1997; Xue et al., 1998), and *gpa2* mutants have decreased heat tolerance (Kraakman et al., 1999). Furthermore, the intrinsic GTPase activity of Gpa2 is stimulated by the RGS protein Rgs2 (Regulator of G-protein signaling) which contains a GAP domain for hydrolysis of GTP to GDP (Versele et al., 1999). The GTP bound form of Gpa2 activates Cyr1 to produce cAMP (Colombo et al., 1998). Hence, production of cAMP by Cyr1 is controlled by two G-proteins that each mediate one branch of a dual glucose-sensing pathway (Figure 1).

### Regulation of PKA Activity

PKA activity is also regulated by additional mechanisms. For example, the Kelch proteins Gpb1 and Gpb2 can bypass adenylyl cyclase and directly bind the catalytic subunits of PKA to promote holoenzyme formation and reduce activity (Lu and Hirsch, 2005; Peeters et al., 2006). This influence of Gbp1 and Gbp2 establishes a mechanism whereby activation of PKA is buffered by the Kelch proteins after an increase in cAMP concentration. Gpb1 and Gpb2 also influence the abundance and phosphorylation state of the PKA regulatory subunit Bcy1 and this protein is also a target of the Sch9 protein kinase (Budhwar et al., 2010, 2011; Zhang et al., 2011). PKA regulation also occurs through interactions with substrates and factors that influence the subcellular localization of the enzyme (Vigil et al., 2004; Galello et al., 2010). For example, a key feature of cAMP signaling in mammalian cells involves the interaction of the holoenzyme with A-kinase anchoring proteins (AKAPs). These proteins act to control the subcellular localization of PKA to influence spatial and temporal specificity. AKAPs are a diverse family of more than 50 members that share the ability to bind to the regulatory subunit of the PKA holoenzyme. In addition to their PKA-binding domain, AKAPs generally contain targeting domains specific for subcellular structures, membranes, or organelles (Søberg and Skålhegg, 2018). Until recently, no AKAPs were described in *S. cerevisiae* or other fungi; however, the subcellular distribution of Bcy1 is regulated by Yak1-dependent phosphorylation of its N-terminal domain, and the Zds1 protein is required for proper cytoplasmic localization of Bcy1 in carbon source-derepressed cells (Griffioen et al., 2000, 2001). Zds1 has been proposed to be an analog of mammalian AKAPs, but an interaction with Bcy1 has not been demonstrated. Galello et al. also identified new proteins that associate with Bcy1 using proteomic and bioinformatic approaches (Galello et al., 2014). Two of these proteins (Ira2 and Hsp60) co-localized with Bcy1 and may participate in determining the subcellular localization of the protein. Ira2 could potentially tether PKA to the Ras complex and the Hsp60 chaperone may localize PKA to mitochondria under conditions of thermal stress.

### Structural Features of the PKA Holoenzyme and Spatial-Temporal Control of cAMP Signaling

The structural features of the PKA holoenzyme and the subunits have been extensively studies in mammalian cells (Taylor et al., 2005; Rinaldi et al., 2010). Particular attention was given to the regulatory subunit which contains an N-terminal region responsible for dimerization and docking to AKAPs (D/D domain), a linker region with an inhibitory site (IS) that occupies the active sites of the catalytic subunits, and two cAMP binding-domains at the C-terminus (Taylor et al., 2005; Rinaldi et al., 2010). The structure and function of the IS and cAMP binding domains have been studied in both mammals and yeast (Kuret et al., 1988; Werner-Washburne et al., 1991; Rinaldi et al., 2010), but scant attention has been paid to the structure of the D/D domain in yeast and fungi. In mammalian cells, the D/D domain is required for the dimerization of the regulatory subunits, and the interaction exposes a hydrophobic groove that serves as a binding platform for AKAPs (Banky et al., 2003). A bioinformatic analysis of sequences from 99 fungi revealed the existence of the canonical D/D domain (1–50 amino acids) in 63 species distributed across four phyla and one subphylum; the 36 fungal sequences lacking the predicted N-terminal region domain belong to the pezizomycotina subphylum (González Bardeci et al., 2016). This study also characterized the structure of Bcy1 and identified a novel tetrameric form leading to a model in which the protein consists of a dimer of dimers via oligomerization of the D/D domain. This organization putatively creates two classical AKAP binding surfaces and additional sites for electrostatic interactions, and suggests that tetramerization of regulatory subunits may allow interaction with a wide range of intracellular partners including AKAPs (González Bardeci et al., 2016) (Figure 1).

In mammalian cells, localized pools or microdomains of cAMP are controlled by cAMP phosphodiesterases (PDEs) to influence the activation of PKA-AKAP complexes at a specific subcellular locations (Taskén and Aandahl, 2004). For example, direct interaction between PDE4D3, the centrosomal PKA-anchoring protein AKAP450, and the PKA regulatory subunit in rat Sertoli cells provides evidence for a spatial-temporal control of cAMP signaling (Taskén et al., 2001; Taskén and Aandahl, 2004). Unlike their mammalian counterparts, the contribution of *S. cerevisiae* PDEs to cAMP microdomains that participate in PKA activation at specific subcellular localizations has yet to be demonstrated. In yeast, two cAMP phosphodiesterases (PDEs), Pde1 and Pde2, are responsible for the hydrolysis of cyclic nucleotides and play an important role in regulating the intracellular levels of cAMP or cGMP. Pde1 is a low-affinity cAMP and cGMP hydrolase, while Pde2 is a high affinity cAMP-specific Mg^2+^-dependent phosphodiesterase (Uno et al., 1983; Suoranta and Londesborough, 1984). Furthermore, PKA exerts a negative feedback loop on PDE activity by phosphorylating Pde1 and regulating Pde2 localization and protein concentration (Ma et al., 1999; Hu et al., 2010). Deletion of *PDE2*, but not *PDE1*, rendered cells sensitive to stressors, such as osmotic and thermal shock, and ROS (Park et al., 2005).

### Transceptor Sensing of Other Nutrients

The cAMP/PKA pathway in *S. cerevisiae* also participates in the sensing of and response to other nutrients including nitrogen sources (e.g., ammonium, amino acids), phosphate, sulfate, and the micronutrients iron and zinc. The underlying mechanisms have been thoroughly reviewed recently and, in general, these nutrients are sensed via transceptor proteins that are thought to activate PKA without influencing cAMP levels (Steyfkens et al., 2018). For example, the Mep2 protein in yeast is mainly responsible for sensing ammonium and activating PKA with downstream effects on nitrogen metabolism and pseudohyphal growth. Similarly, the ammonium sensors Atm1, 2 have been identified in *C. neoformans* and other fungal pathogens (Rutherford et al., 2008a,b; Lee et al., 2012; Steyfkens et al., 2018). Other yeast transceptors may be relevant to fungal pathogenesis including the general amino acid permease Gap1 (Cain and Kaiser, 2011). This protein mediates amino acid activation of PKA to influence mobilization of trehalose and glycogen, the response to stress, and ribosomal protein expression. Additionally, the Pho84 transceptor mediates activation of PKA in response to phosphate, Sul1 and Sul2 control the response to sulfate, and Ftr1 and Zrt1 are responsible for the responses to iron and zinc, respectively (Popova et al., 2010; Kankipati et al., 2015; Schothorst et al., 2017). As discussed further below, the mechanisms by which yeast senses amino acids, phosphate, iron and zinc may be particularly relevant because studies to date have implicated these nutrients in the virulence of *C. neoformans*.

## The camp/Pka Pathway In *C. neoformans*

### Identification and Characterization of Pathway Components

**Figure 2:**
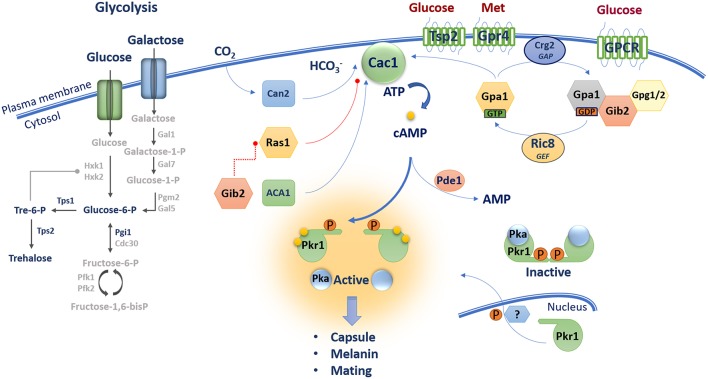
The cAMP/PKA pathway in *C. neoformans* contains core components including a G-protein coupled receptor Gpr4 [activated by methionine (Met)], a Gα protein Gpa1, a Gβ-like/RACK1 homolog Gib2, Gγ proteins Gpg1 and Gpg2, an RGS protein Crg2 and a GEF Ric8. These proteins along with CO_2_/HCO_3_- regulated by the carbonic anhydrase Can2 influence the activity of adenylyl cyclase Cac1 and the production of cAMP. Gib2 potentially promotes cAMP levels through the inhibition of Ras1 functions on Cac1. Unknown GPCRs and the tetraspanin protein Tsp2 sense external glucose and may participate in cAMP/PKA activation. The level of cAMP influences the dissociation of the catalytic (Pka1 and Pka2) and regulatory (Pkr1) subunits of PKA leading to activation of the kinase and downstream signaling. The phosphodiesterase Pde1 influences intracellular cAMP levels.

The core features and components of the cAMP/PKA pathway are generally conserved between *S. cerevisiae* and *C. neoformans* (Figure 2). However, in *C. neoformans*, the cAMP/PKA pathway has been adapted to connect nutrient sensing to downstream functions and other signaling pathways that remodel the cell surface for deployment of virulence factors such as capsule and melanin. In terms of the nutrients sensed by *C. neoformans*, there are hints that glycolysis is connected to cAMP/PKA signaling, although direct connections have yet to be established. For example, mutants impaired in glycolysis due to defects in the genes for hexokinase are attenuated for virulence in animal models of cryptococcosis (Price et al., 2011). Additionally, deletion of the gene encoding phosphoglucose isomerase Pgi1 in *C. neoformans* abolished capsule and melanin formation, and addition of exogenous cAMP restored these phenotypes (Zhang et al., 2015). Furthermore, the genes for trehalose synthesis, *TPS1*, and *TPS2*, are required for virulence factor elaboration (i.e., melanin, capsule, thermotolerance) and control protein secretion, mating, and cell wall integrity in the related *Cryptococcus* species, *C. gattii* (Ngamskulrungroj et al., 2009). These results suggest that glycolysis is likely involved in cAMP/PKA activation, although further studies are needed, including focused attention on the role of fructose-1,6-bisphosphate.

With regard to GPCRs that potentially sense extracellular glucose, seven candidate proteins (Ste3/Cpr, Cpr2, and Gpr1–5) have been identified in *C. neoformans*, and Gpr4 has been characterized in detail because of its sequence similarity to the *Dictyostelium discoideum* cAMP receptor cAR1 and yeast Gpr1 (Xue et al., 2006). Mutants of *GPR4* exhibit a reduced capsule phenotype and are impaired in mating, but maintain the ability to produce melanin and cause disease in a mouse model of systemic infection (Xue et al., 2006). Interestingly, Gpr4 appears to respond to exogenous methionine rather than glucose. That is, analysis of cAMP levels showed that *gpr4* mutants are still able to respond to glucose, but not to methionine; methionine also triggers Gpr4 receptor internalization (Xue et al., 2006). A putative tetraspanin protein Tsp2 is also proposed to be a candidate glucose sensor based in part on the discovery that this protein negatively regulates melanin production, a phenotype that is repressed by glucose (Li et al., 2012). The Tsp2 protein also influences capsule formation, and mutant phenotypes can be rescued by addition of cAMP. Several components of heterotrimeric G proteins have been identified in *C. neoformans* that may function downstream of GPCRs, and possibly Tsp2. These include three Gα subunits (Gpa1, Gpa2, and Gpa3), one Gβ protein (Gpb1), one Gβ-like protein (Gib2) and two Gγ proteins (Gpg1 and Gpg2). Three G proteins (Gpa2, Gpa3, and Gpb1) were found to be involved only in mating (Wang et al., 2000; Li et al., 2007), Gpa1 and Gib2 appear to be specific to the cAMP/PKA pathway, and Gpg1 and Gpg2 are shared between the mating and cAMP/PKA pathways (Alspaugh et al., 1997; Wang et al., 2000; Palmer et al., 2006; Hsueh et al., 2007; Li et al., 2007). Gpa1 interacts with Gpr4 and regulates mating, capsule elaboration, melanin and cAMP production. In fact, the initial connection between cAMP/PKA signaling and virulence in *C. neoformans* came from an analysis of Gpa1 (Alspaugh et al., 1997). Addition of exogenous cAMP suppresses *gpa1* mutant phenotypes suggesting that Gpa1 participates in mating and virulence factor elaboration via the cAMP-PKA pathway (Alspaugh et al., 1997, 2002; D'Souza et al., 2001; Xue et al., 2006). Gpa1 regulates the transcript levels for multiple genes involved in capsule and melanin production thus providing further evidence that these traits are regulated by the cAMP/PKA pathway (Pukkila-Worley et al., 2005). Gpa1 is also involved in mating but does not interact with Gbp1 in a yeast two-hybrid assay; addition of cAMP restores mating in a *gpa1* mutant, but not in a *gbp1* mutant (Wang et al., 2000). Taken together, Gpr4 may be one of several GPCRs coupled to Gpa1 to activate the cAMP-PKA pathway, but additional studies are needed to identify and characterize a specific glucose-sensing GPCR, or alternative sensors such as Tsp2.

An additional component of the cAMP/PKA pathway, the non-canonical Gβ-like/RACK1 protein homolog Gib2, was identified in a yeast two-hybrid screen using Gpa1 as bait (Wang et al., 2000). Gib2 contains a seven WD-40 repeat motif and shares 70% identity with the mammalian G-like/RACK1 protein GNB2L1. Overexpression of Gib2 suppresses capsule and melanin defects in *gpa1* mutants suggesting that the protein promotes cAMP signaling downstream of Gpa1. However, disruption of Gib2 did not cause defects in capsule, melanin, or cAMP production (in contrast to the *gpa1* mutant), but rather the mutant displayed a growth delay at 37°C and a virulence defect (Palmer et al., 2006; Wang et al., 2014). Gib2 interacts with ~50 proteins including proteins involved in translation and ribosome composition. Additionally, a two-dimensional differential gel electrophoresis (DIGE) analysis revealed that homologs of the heat shock protein Hsp70/71 (e.g., Ssa1) were increased in abundance in a *gib2* mutant. Overall, these results suggest that Gib2 modulates multiple cellular processes such as ribosomal biogenesis, translation and stress responses that are important for the growth and virulence of *C. neoformans* (Palmer et al., 2006; Shen et al., 2010; Wang et al., 2014; Ero et al., 2015; Bruni et al., 2017). The Gγ proteins Gpg1 and Gpg2 (identified by sequence homology) were also found to bind Gib2 although their roles in cAMP signaling are not fully understood (Palmer et al., 2006). It has been proposed that Gib2 binding to Gpa1 and Gpg1/Gpg2 may make a structural contribution to the canonical heterotrimeric Gpa1 protein complex. Gpg1/Gpg2 were also found to bind the Gβ protein Gpb1, which is not involved in cAMP/PKA signaling pathway but is important for mating (Wang et al., 2000; Hsueh et al., 2007). Additional proteins that influence the activities of heterotrimeric G proteins have been identified in *C. neoformans* including guanine nucleotide exchange factors (GEF) and regulators of G protein signaling (RGS) that stimulate the GTPase activity of Gα proteins. For example, the Ric8 (resistance to inhibitors of cholinesterase 8) homolog exhibits guanine nucleotide exchange factor (GEF) activity to promote GDP-GTP exchange. Ric8 interacts with Gpa1 and Gpa2, but not Gpa3. Similar to a *gpa1* mutant, *ric8* mutants had a smaller capsule and reduced melanin formation, and these phenotypes were rescued by cAMP. Moreover, a *ric8* mutant showed reduced mating but was enhanced in its ability to induce a pheromone response in a mating partner. Thus Ric8 is involved in mating (Gong et al., 2014). The RGS proteins Crg1 and Crg2 were also found to regulate G-proteins and to display GAP activity toward Gα subunits. Crg1 interacts with Gpa2 and Gpa3 of the mating pathway (Li et al., 2007; Shen et al., 2008). Cgr2 interacts with all of the Gα subunits (Gpa1, Gpa2, and Gpa3) and the GPCR Gpr4, and Crg2 may therefore form a protein complex with Gpa1 and Gpr4 that is important for cAMP/PKA signaling (Hsueh et al., 2007; Shen et al., 2008; Xue et al., 2008).

### Characterization of Adenylyl Cyclase, Ras Proteins, and the Subunits of PKA

The adenylyl cyclase Cac1 catalyzes production of cAMP in *C. neoformans* (Alspaugh et al., 2002). Similar to *gpa1, cac1* mutants fail to produce capsule and melanin, and addition of exogenous cAMP restores wild-type (WT) phenotypes. Gpa1 is thought to function downstream of Cac1 in the cAMP/PKA pathway although a direct interaction of Cac1 and Gpa1 has not been demonstrated (Alspaugh et al., 2002). An interaction between Cac1 and the adenylyl cyclase-associated protein Aca1 was found in a yeast two-hybrid assay, and confirmed by bimolecular fluorescence complementation and coimmunoprecipitation (Bahn et al., 2004; So et al., 2017). Aca1 appears to function in parallel with Gpa1 to induce Cac1 activity, and deletion of *ACA1* results in defects in mating, capsule formation, and melanin production; these phenotypes are remediated by addition of exogenous cAMP. Furthermore, the *aca1* mutant was defective in cAMP production after glucose readdition, but maintained initial basal levels of cAMP. This basal level was further reduced to that of the *cac1* mutant in a *aca1 gpa1* double mutant, suggesting that Aca1 plays a role in glucose-induced production of cAMP in a manner distinct from that of Gpa1 (Bahn et al., 2004). Activation of Cac1 can also be achieved by CO_2_ conversion to bicarbonate accelerated by the carbonic anhydrase Can2, suggesting that the cAMP/PKA pathway can be triggered by additional stimuli that may be relevant to the mammalian host environment. An analysis of transcriptome changes revealed that a majority of Can2-dependent genes are involved in the response to environmental stress (Mogensen et al., 2006; Kim et al., 2010).

Genes encoding the Ras proteins Ras1 and Ras2 have been characterized in *C. neoformans*, and found to play distinct and shared roles in filamentation and mating as well as promoting growth under thermal stress (Alspaugh et al., 2000; Waugh et al., 2002; Nichols et al., 2007; Ballou et al., 2013). Interestingly, Ras1 interacts with Cac1 through its putative RA domain and with Gib2 as demonstrated in a yeast two-hybrid assay, suggesting that Gib2 could regulate cAMP signaling via Ras1 and Cac1 (Wang et al., 2014). Genetic evidence supports this possibility because capsule size and melanin production are unchanged in a *ras1* mutant, whereas these factors are abolished in a *cac1* background. Furthermore, an enlarged capsule and restored melanin production were observed in a *gpa1 ras1* double mutant (suppressing *gpa1* phenotypes) indicating that Ras1 acts downstream of Gpa1 and negatively regulates Cac1. A specific role for Gib2 in the regulation of Ras1 has yet to be demonstrated, but Gib2 may influence cAMP levels by impairing the inhibitory activity of Ras1 on Cac1 in the absence of Gpa1 (Wang et al., 2014). Ras1 functions in multiple cellular processes in *C. neoformans* including thermotolerance, cell polarity, hyphal morphogenesis and cell cycle regulation (Nichols et al., 2007; Ballou et al., 2013). Therefore, it is possible that the inhibitory role of Ras1 on Cac1 is stimulated under conditions other than glucose sensing. In fact, transcriptome analysis demonstrates that *aca1, gpa1* and *cac1* mutants display similar patterns, whereas a *ras1* mutant exhibits a distinct pattern of gene expression. A number of environmental stress response genes were modulated differently, suggesting that the Ras1 signaling pathway is largely independent of the cAMP/PKA pathway in *C. neoformans* (Maeng et al., 2010).

The genes *PKA1* and *PKA2* encode the catalytic subunits of PKA in *C. neoformans*, and the *PKR1* gene encodes the regulatory subunit. In a strain of the A capsule serotype, a mutation in *PKA1* abolishes mating, melanin formation, and capsule production; in contrast, a mutation of *PKR1* results in cells with an enlarged capsule and the ability to produce melanin. Deletion of *gpa1* in a *pkr1* mutant restores capsule and melanin production, and a double mutant *pka1 pkr1* behaves like a *pka1* mutant. These results indicate that Pka1 and Pkr1 function downstream of Gpa1 and that Pka1 functions downstream of Pkr1 regulatory subunit. Interestingly, intracellular levels of cAMP were drastically elevated in a *pka1* mutant as compared to the WT strain, suggesting that PKA may function to limit cAMP production (D'Souza et al., 2001). Pka2 was found to have no involvement in capsule and melanin production in serotype A strains (Hicks et al., 2004). A transcriptional analysis of *pka1* and *pkr1* mutants revealed that the cAMP/PKA pathway influences transcript levels for genes involved in capsule and cell wall synthesis, secretion, iron transport, tricarboxylic acid cycle (TCA), phospholipid synthesis, glycolysis, ribosome biogenesis, and heat shock response (Hu et al., 2007). Similar *to S. cerevisiae*, control of cAMP is also mediated by phosphodiesterases in *C. neoformans*. Genes for two enzymes (Pde1 and Pde2) were identified, but Pde2 has no apparent role in regulating intracellular cAMP levels because a *pde2* deletion only resulted in subtle mutant phenotypes. However, Pde1 clearly participates in the cAMP/PKA pathway because a *pde1* mutation rescued phenotypes of the *gpa1* mutant (i.e., capsule and melanin production). Additionally, intracellular levels of cAMP were increased in *pde1, pde1 pde2, pde1 pka1*, and *pde1 pde2 pka1* mutants (Hicks et al., 2005; Wang et al., 2014). Overall, the core features of the cAMP/PKA pathway in *C. neoformans* mirror the pathway in *S. cerevisiae* (Figures 1, 2), and the challenge, as discussed below, is to understand the targets of pathway regulation that influence the ability of the fungus to cause disease.

## camp/Pka Signaling And Cryptococcal Virulence

The importance of the cAMP/PKA pathway in the ability of *C. neoformans* to cause disease is illustrated by the finding that mutants defective in core components (i.e., Gpa1, Cac1, Aca1, and Pka1) have attenuated virulence in a mouse model of cryptococcosis (Alspaugh et al., 1997, 2002; D'Souza et al., 2001; Bahn et al., 2004). Virulence defects associated with the cAMP/PKA pathway likely arise from problems in elaborating virulence factors including capsule, enzymes such as proteases, urease, superoxide dismutase, and laccase, as well as altered metabolic functions needed for proliferation (reviewed by O'Meara and Alspaugh, 2012; Choi et al., 2015). The downstream targets of PKA are therefore clearly important for understanding connections between environmental sensing and the ability of *C. neoformans* to survive and proliferate in mammalian hosts. As outlined below, the molecular mechanisms that link nutrients or environmental conditions to the pathway and downstream targets are actively being investigated.

### Connections Between cAMP/PKA Signaling, Iron, and Phosphate

**Figure 3:**
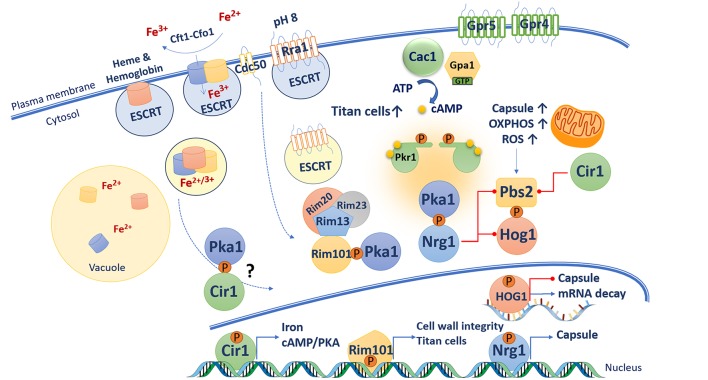
Connections between the cAMP/PKA pathway and virulence-related functions in *C. neoformans*. A variety of functions act at the cell surface to sense potential signals of relevance to mammalian hosts. These include iron sensing via the high affinity uptake system (Cft1-Cfo1), the ESCRT pathway and master iron regulator Cir1. The ESCRT pathway also participates in the pH-response RIM pathway. In response to alkaline pH, Rim101 is phosphorylated by PKA, cleaved by the protease Rim13, and relocalized to the nucleus where it regulates the expression of genes involved in cell wall remodeling. The cAMP/PKA and HOG pathways have opposing influences on capsule formation. In presence of glucose, cAMP activates PKA, which in turns phosphorylate the transcription factor Nrg1 that promotes expression of capsule synthesis gene and represses transcription of *PBS2* and *HOG1*. Capsule enlargement requires increase mitochondrial oxidative phosphorylation activity, which generate increase of reactive oxygen species (ROS). ROS triggers Hog1 activation via its phosphorylation by Pbs2. Phosphorylated Hog1 translocates to the nucleus and reduces capsule biosynthesis by potentially accelerating mRNA degradation. The cAMP/PKA and RIM pathways as well as GPCR Gpr5 are required for titan cell elaboration.

The polysaccharide capsule is a major virulence factor for *C. neoformans* as demonstrated by the observation that deletion of genes for capsule production (e.g., *CAP59*) abolish disease in a mouse model of cryptococcosis (Chang and Kwon-Chung, 1994). Capsule elaboration is the result of a highly regulated process where nutrient sensing pathways and a transcription factor network respond to a variety of signals relevant to the host environment (Maier et al., 2015; Gish et al., 2016). For example, a long-standing observation is that reduced iron availability results in enhanced capsule production (Vartivarian et al., 1993), and this suggests that *C. neoformans* responds to the iron withholding that is a key aspect of host nutritional immunity (Hood and Skaar, 2012). In fact, regulation of capsule involves both the cAMP/PKA pathway and the iron regulator Cir1, GATA-type zinc finger transcription factor (D'Souza et al., 2001; Jung et al., 2006; Attarian et al., 2018) (Figure 3). Transcriptome analyses revealed that Cir1 regulates genes encoding components of the cAMP/PKA pathway (e.g., Grp4 and Cac1) and phospholipases, as well as functions involved in melanin and capsule formation, and cell wall and membrane synthesis (Jung et al., 2006). In addition, mutants lacking components of the CCAAT-binding transcription factor complex, Hap3 and Hap5, are defective in capsule production (Jung et al., 2010). These proteins, and the regulatory subunit HapX, are also involved in the regulation of iron homeostasis and have a regulatory interaction with Cir1 and the pH-responsive transcription factor Rim101 (discussed below). Additional connections between iron and the cAMP/PKA pathway include regulation by PKA of the genes encoding the high affinity iron permease Cft1 and the siderophore transporter Sit1 (Hu et al., 2007; Tangen et al., 2007). Pka1 also influences the intracellular localization of the ferroxidase Cfo1 that is thought to work with Cft1 in high affinity iron uptake (Jung et al., 2009). Furthermore, a proteomic analysis of secreted proteins in a strain with regulated expression of Pka1 identified regulation of the cytokine-inducing glycoprotein Cig1 that is associated with heme uptake (Cadieux et al., 2013; Geddes et al., 2015). Heme trafficking in *C. neoformans* also involves the endosomal sorting complex required for transport (ESCRT) machinery, and functional analyses have demonstrated roles in iron acquisition and capsule elaboration. The ESCRT machinery act downstream of the cAMP/PKA pathway because a defect in the ESCRT protein Vps23 reduces the otherwise enlarged capsule of a *pkr1* mutant (Hu et al., 2015). Altogether, these findings strongly suggest a connection between the cAMP/PKA pathway, iron sensing and acquisition, and regulation of capsule elaboration. The details of the mechanisms that connect iron sensing and the cAMP/PKA pathway are not known, but one can hypothesized that changes in intracellular iron sensing and/or in the iron labile pool trigger multiple signaling pathways (including the cAMP/PKA pathway) in order to adapt and survive.

Given that the iron permease Ftr1 is a transceptor that activates PKA in *S. cerevisiae*, it is possible that the iron permease Cft1 may play a similar role to connect iron and cAMP/PKA signaling in *C. neoformans*. This idea requires further study and possible roles as iron transceptors should also be considered for two other potential iron permeases Cft2 and Cft3 (Jung et al., 2008; Han et al., 2012). The possibility of transceptors that influence capsule also extends to phosphate sensing. As mentioned above, Pho84 is a transceptor for phosphate that activates PKA in *S. cerevisiae* (Samyn et al., 2012). Three phosphate transporters have been characterized in *C. neoformans* (Pho84, Pho840, and Pho89) and a triple mutant is attenuated for capsule formation and for virulence in mice (Kretschmer et al., 2014). Additionally, a *pkr1* mutant (and a *cir1* mutant) showed reduced growth on phosphate-limited medium thus suggesting a connection with the cAMP/PKA pathway. The *pka1* and *pkr1* mutants were also impaired in the production of polyphosphate (polyP), and exogenous cAMP was found to enhance polyP levels in WT cells. Other potential transceptors have yet to be characterized and tested for connections to PKA in *C. neoformans*, although candidates for amino acids and zinc have been identified (Do et al., 2016; Calvete et al., 2019).

### cAMP/PKA Regulation of the pH-Responsive RIM Pathway

The cAMP/PKA pathway in *C. neoformans* also activates the pH response regulator Rim101 that is responsible for sensing extracellular pH as part of the RIM pathway (O'Meara et al., 2010). The Rim pathway is under active investigation in *C. neoformans* with the identification of homologs of core components known from *S. cerevisiae* and other fungi as well as novel components identified in genetic screens (O'Meara et al., 2010, 2014; Ost et al., 2015, 2017; Brown et al., 2018; Pianalto et al., 2018). Initial activation of the RIM pathway in *C. neoformans* involves extracellular sensing of pH (neutral and alkaline) by a 7-transmembrane domain protein sensor Rra1 (Ost et al., 2015). Once activated, Rra1 likely undergoes endocytosis with the participation of the flippase component Cdc50 and the ESCRT machinery (e.g., Vps23 and Snf7), leading to cleavage of Rim101 by the protease Rim13 (Ost et al., 2015). Cdc50 is also required for iron acquisition, membrane integrity, and survival at alkaline pH, and the protein is involved in activation, cleavage, and nuclear localization of Rim101 as *cdc50* mutants are delayed in these processes (Hu et al., 2017; Brown et al., 2018). Rim101 cleavage requires PKA phosphorylation and the scaffold protein Rim20 (which interacts with the protease Rim13), and together these factors regulate the nuclear localization, activation, and processing of Rim101. Inactivation of the PKA phosphorylation site by site-directed mutagenesis results in mislocalization of Rim101 and reduced capsule size in an otherwise hyper-encapsulated *pkr1* mutant. These results further support the idea that capsule elaboration (i.e., cell surface attachment) is modulated by Rim101 via its phosphorylation by Pka1 (O'Meara et al., 2010; Ost et al., 2015). Interestingly, the Gpa1 Gα protein in the cAMP/PKA pathway is not required for Rim101 activation suggesting that signaling to PKA must be through another mechanism (Ost et al., 2015).

Cleaved Rim101 translocates to the nucleus to regulate the expression of genes encoding functions for cell wall integrity, metal homeostasis, and capsule attachment to the cell wall. Comparative transcriptional analysis demonstrates a strong similarity in the impact of PKA and Rim101 on gene expression (O'Meara et al., 2014). The influence of Rim101 on the cell wall is important for regulating surface exposure of features such as chitin and chitosan that provoke a damaging inflammatory response (Ost et al., 2017). Other studies further emphasize the importance of cell wall integrity (CWI) and cAMP/PKA signaling in the virulence of *C. neoformans*. For example, disruption of genes encoding kinases involved in the cell wall integrity pathway (i.e., Bck1, Mkk2, and Mpk1) revealed significantly less intracellular cAMP, and a smaller capsule phenotype, under inducing conditions (Donlin et al., 2014). Addition of exogenous cAMP protects cells against cell wall stressors and restores capsule formation. These results provide evidence of cross talk between the CWI and cAMP/PKA pathways for the regulation of cell wall and capsule production (Donlin et al., 2014). Another example comes from the recent characterization of the *Cryptococcus* Vilse homolog (Cvh1), a protein that is highly homologous to the Rho GAP-containing Vilse protein in *Drosophila melanogaster* (Rahim et al., 2017). This putative RhoGAP protein is important for capsule and melanin formation, and *cvh1* mutants exhibit hypersensitivity to osmotic stress, heat shock and cell wall stressors, suggesting a role in regulatory circuits that govern CWI (Rahim et al., 2017). Interestingly, addition of exogenous cAMP restores melanin but not capsule formation to the *cvh1* mutant.

### Cross Talk Between the High Osmolarity Glycerol (HOG) Response and cAMP/PKA Pathways

The HOG pathway in *C. neoformans* plays a central role in the response to various stresses (e.g., osmotic, temperature, and oxidative stresses), and virulence factor elaboration (i.e., capsule and melanin formation). The pathway and its role in the adaptation of cells to increased osmolarity and other stress conditions are well-characterized in *S. cerevisiae* (Brewster and Gustin, 2014). In yeast, the phosphorylation and activity of the Hog1 MAPK (Mitogen Activated Protein Kinase) is controlled by two branches activated by the membrane sensors Sln1 or Sho1. The pathways activated by these sensors converge on the MAP kinase kinase (MAPKK) Pbs2. Pbs2 dually phosphorylates and activates Hog1 which is then imported into the nucleus to influence the expression of genes that function to mediate the response to stress (Hohmann, 2009). Deletion of the ortholog encoding Hog1 in a strain of *C. neoformans* with the A capsular serotype results in an enlarged capsule and increased melanin pigmentation. A mutant lacking Pbs2 shares the phenotypes of the *hog1* mutant (Bahn et al., 2005). Deletion of the genes *gpa1, cac1* or *pka1* in a *hog1* mutant background resulted in acapsular phenotypes, and the double mutants also had restored melanin production compared with the ablated pigment formation in the *gpa1, cac1*, or *pka1* single mutants. These results clearly demonstrate a connection with the cAMP/PKA pathway. Transcriptome analyses reveal that Hog1 and Pbs2 are transcriptionally repressed by the transcription factors Cir1 and Nrg1 (Haynes et al., 2011). Nrg1 is a C_2_H_2_ zinc finger transcription factor that was identified in a microarray experiment and shown to be regulated by cAMP and glucose. Disruption of *NRG1* results in a reduced capsule phenotype and delayed mating, and mutation of the putative PKA phosphorylation site resulted in a reduced capsule similar to a *ngr1* deletion mutant (Cramer et al., 2006). This result suggests that phosphorylation of Nrg1 by Pka1 is required for Nrg1 activity. Furthermore, Nrg1 controls the expression of multiple genes involved in carbohydrate metabolism as well as the *UGD1* gene encoding for a UDP-glucose dehydrogenase required for capsule biosynthesis (Moyrand and Janbon, 2004; Cramer et al., 2006). Therefore, connections between the cAMP/PKA and the HOG pathway may be mediated in part by Nrg1, although additional work is needed to understand the complexities of the interplay between the pathways.

A further complexity for the interactions of the cAMP/PKA and Hog1 pathways involves their control of a post-transcriptional response to glucose availability (Banerjee et al., 2016). In the host, *C. neoformans* must adapt to differences in glucose availability that vary from a limited amount in the lungs to more glucose-rich environments in the blood and brain. Glucose availability impacts ribosome biogenesis and translation by modulating mRNA decay. In the glucose-limited condition, generation of oxidative stress occurs as demonstrated by Hog1-dependent increases in the levels of the catalase Cat3 and the superoxidase Sod2. Oxidative stress triggers Hog1 activation that in turn mediates ribosomal protein (RP) mRNA degradation. In the glucose-replete condition, RP transcripts are induced and stabilized by Pka1 to support protein synthesis and growth. These results suggests that control at the level of mRNA stability by the opposing actions of Hog1 and PKA is an adaptative molecular mechanism to regulate the abundance of RP transcripts and ribosome biogenesis (Banerjee et al., 2016). In the context of oxidative stress and reactive oxygen species, mitochondrial functions may also be connected to signaling pathways for the regulation of capsule synthesis (Trevijano-Contador et al., 2017). Capsule growth was observed to correlate with increased mitochondrial membrane potential and higher production of reactive oxygen species. Inhibition of the respiratory chain complex III and the alternative oxidase Aox1 resulted in capsule reduction (Trevijano-Contador et al., 2017). Taken together, the recent studies reveal complex connections between metabolism (glucose sensing, mitochondrial oxidative phosphorylation), capsule synthesis, cellular proliferation, and the cAMP/PKA and Hog1 pathways.

### Role of the cAMP/PKA Pathway in Proteostasis

Although there has been some success in identifying transcription factors that are known or potential targets of PKA (e.g., Rim101), it has generally been challenging to identify targets of PKA phosphorylation. To address this challenge, a quantitative proteomics was recently employed with the strains developed by Choi et al. (2012) that express *PKA1* under the control of the glucose-repressed, galactose-activated *GAL7* promoter. The proteomes of the engineered strains were compared with those of WT cells grown in matched carbon source media (with either glucose or galactose). Both the secretome and the intracellular proteome were characterized and the latter analysis revealed an influence of elevated *PKA1* expression on proteins associated with translation, the proteasome, metabolism, amino acid biosynthesis and virulence (Geddes et al., 2015, 2016). The connection between PKA and ubiquitin-proteasome pathway was investigated further by examining the influence of the proteasome inhibitor bortezomib (BTZ) on PKA-related phenotypes. In particular, inhibition of proteasome function by BTZ was sufficient to reduce capsule size and to inhibit the proliferation of the strains with altered PKA function. In the latter situation, strains predicted to have high PKA1 expression and/or high PKA activity were hypersensitive to BTZ. Interestingly, treatment of BTZ and cAMP together inhibited the proliferation of P_*GAL*7_*:PKA1*, and WT strains as well as a *pkr1* mutant. Overall, these results suggest that activation of PKA influences proteostasis and that the proteasome influences capsule elaboration (Geddes et al., 2016).

The connection between the proteasome and capsule was reinforced further by investigations on the influence of lithium on capsule formation. A previous study revealed that lithium treatment reduces capsule size in WT and *pkr1* strains, and impairs the proliferation of a *pka1* mutant (Hu et al., 2007). A screen of the collection of deletion mutants for strains with altered sensitivity to lithium identified several components of the ubiquitin-proteasome system (Liu et al., 2008; Geddes et al., 2016; Mayer et al., 2018). Interestingly, the combination of lithium and BTZ appeared to reduce capsule size to a greater extent than either drug alone (Mayer et al., 2018). Therefore, it may be possible to exploit drug combinations as an anti-virulence approach to treat cryptococcosis (Vu et al., 2019).

### The cAMP/PKA Pathway Participates in Titan Cell Elaboration

*C. neoformans* undergoes a remarkable morphological adaptation during proliferation in host lung tissue with the formation of a subpopulation (~20%) of enlarged cells called titan cells (50 to 100 μm vs. the typical size of 5–8 μm). Titan cells have several distinguishing properties in addition to their large size, including increased ploidy, cell wall changes and a condensed capsule (Okagaki et al., 2010; Zaragoza et al., 2010; García-Rodas et al., 2018). Titan cells also influence the interaction with host cells and enhance virulence, have increased drug resistance, and altered exposure of pathogen associated molecular patterns (Okagaki et al., 2010; Crabtree et al., 2012; Okagaki and Nielsen, 2012; Gerstein et al., 2015; Mukaremera et al., 2018). The cAMP/PKA pathway is involved in the formation of titan cells based on the initial finding that mutants lacking the adenylyl cyclase Cac1, but not Ras1, did not form titan cells (Zaragoza et al., 2010). In addition, a constitutively active *GPA1* mutant allele or a mutation in *PKR1* gene resulted in a significant increase in titan cell formation, indicating that cAMP/PKA pathway is required for titan cell elaboration (Okagaki et al., 2011; Hommel et al., 2018). A putative GPCR Gpr5, which is a homolog of Gpr4, was demonstrated to be required for titan cell production, however a *gpr5* mutation did not result in defects in capsule and melanin formation, or changes in thermotolerance, suggesting that Grp5 does not relay signals to the cAMP/PKA pathway (Zaragoza et al., 2010; Okagaki et al., 2011). More recent work suggests that Gpr4, Gpr5, and the Ste3a pheromone receptor may signal through Gpa1, the cAMP/PKA pathway and Rim101 to regulate titan cell formation (Dambuza et al., 2018). Additionally, overexpression of *PKA1* caused increase in cell size and ploidy, an hallmark phenotype of titan cells as they replicate their DNA asymmetrically (Choi et al., 2012; Gerstein et al., 2015). Furthermore, activation of the Rim101 pathway is required for titan cell development as mutant in *RIM101* produced very few titan cells (Okagaki et al., 2011).

Considerable insights into the formation of titan cells were provided by three recent papers that define *in vitro* conditions to generate these cells (Dambuza et al., 2018; Hommel et al., 2018; Trevijano-Contador et al., 2018). These papers identified a variety of signals that influence cell enlargement for *C. neoformans* and further connect the cAMP/PKA pathway to the process. For example, CO_2_ is an important factor for titan cell formation, and CO_2_ may be important *in vivo* because capsule formation is induced in response to the gas in the lungs (Bahn et al., 2005; Mogensen et al., 2006; Kim et al., 2010). Carbonic anhydrases Can1 and Can2 converts CO_2_ into HCO_3_-, and Can2 activates the adenylyl cyclase Cac1. Interestingly, deletion of *CAN1*, but not *CAN2* results in enhancement of titan cell production. It was postulated that the absence of Can1 might boost Can2 activity on Cac1 (Trevijano-Contador et al., 2018). Cell enlargement has been also observed in response to phosphatidylcholine, components of peptidoglycan, macrophages, amoeba, and the wax moth *Galleria mellonella* suggesting that titan cell formation may be triggered by host derived signals. In this regard, phospholipids such as phosphatidylcholine have been demonstrated to influence cell and capsule enlargement, and phospholipid sensing is related to the cAMP/PKA pathway by an unknown mechanism (Chrisman et al., 2011; García-Rodas et al., 2011). It is known however that loss of a PKA-regulated gene (*OVA1)* encoding a putative phosphatidylethanolamine-binding protein in a *pka1* background restores capsule and melanin production. Ova1 may therefore link phospholipid sensing to cAMP signaling pathway (Hu et al., 2007). It is also interesting that muramyl dipeptide from peptidoglycan triggers titan cell formation suggesting a potential impact of bacteria on *C. neoformans* morphology, perhaps during interactions between the fungus and the lung microbiota (Dambuza et al., 2018).

## Conclusions

The cAMP/PKA pathway in *C. neoformans* plays a well-documented role in sensing nutrients to regulate adaptation to host-relevant conditions and the elaboration of virulence factors. The pathway mediates adaptation through its extended connections with other functions and pathways that regulated iron homeostasis, the RIM pH response pathway, and the HOG pathway. The cAMP/PKA pathway also plays an important role in orchestrating cell surface remodeling and titan cell development. However, molecular mechanisms underlying these phenotypic changes have yet to be characterized. Recent findings in the *S. cerevisiae* shed light on mechanisms of nutrient sensing via glycolysis, and suggest that the role of fructose-1,6-bisphosphate in glucose sensing should be investigated in *C. neoformans*. Additionally, considerable effort is needed to identify direct targets of phosphorylation by PKA and to integrate the functions of these targets into the emerging framework of cAMP/PKA regulation of virulence (Figure 3). There is also a clear need to identify the sensors at the cell surface that transmit nutritional information to the pathway and activate PKA. Along these lines, it will be important to test whether the rapidly growing variety of emerging signals for capsule enlargement and titan formation impact PKA activation. These signals include iron and zinc, phosphate, phospholipids, quorum sensing molecules (pantothenic acid, QSP1 peptide), peptidoglycan and other bacterial products. The hope is that an understanding of key nutritional signals, and subsequent mechanisms of sensing and adaptation may identify opportunities for new antifungal therapies.

## Author Contributions

All authors listed have made a substantial, direct and intellectual contribution to the work, and approved it for publication.

### Conflict of Interest Statement

The authors declare that the research was conducted in the absence of any commercial or financial relationships that could be construed as a potential conflict of interest.
